# Evaluation of a mobile health–enabled hierarchical care model for hypertension management and health-related quality of life among older adults: a prospective pre–post intervention study

**DOI:** 10.3389/fpubh.2026.1964536

**Published:** 2026-10-19

**Authors:** Xiaolei Ma, Kangcheng Li, Dingrong Zheng, Yingying Song, Jian Lin, Ling Chen

**Affiliations:** 1 Shenzhen Longhuan District Center for Disease Prevention and Control, Shenzhen, Guangdong, China; 2 Department of Laboratory, Shenzhen Second People’s Hospital, Shenzhen, Guangdong, China; 3 Department of Medical Insurance and Pricing Management, Shenzhen Bao’an District Central Hospital, Shenzhen, Guangdong, China; 4 School of Public Health and Management, Guangzhou University of Chinese Medicine, Guangzhou, Guangdong, China; 5 Department of Gastrointestinal Surgery, Shenzhen Hospital, Southern Medical University, Shenzhen, Guangdong, China; 6 School of Nursing, Southern Medical University, Guangzhou, Guangdong, China

**Keywords:** digital health, health-related quality of life, hierarchical care, hypertension management, mobile health, older adults, regional medical consortiums

## Abstract

**Background:**

Hypertension remains a major public health challenge among older adults, particularly in the context of population ageing and increasing demand for long-term chronic disease management. Although mobile health (mHealth) technologies have demonstrated potential in supporting hypertension self-management, evidence regarding their integration with hierarchical healthcare delivery systems and their association with patient-reported health outcomes remains limited. This study evaluated changes in blood pressure control and health-related quality of life (HRQoL) following implementation of a mHealth-enabled hierarchical care model within regional medical consortia.

**Objective:**

This study aimed to assess changes in blood pressure indicators and HRQoL following implementation of a mobile health–enabled hierarchical diagnosis and treatment education program (mHealth-HDTEP) among older adults with hypertension.

**Methods:**

A prospective, single-arm, pre-post evaluation study was conducted from January to December 2025 among older adults with hypertension recruited from regional medical consortia in Shenzhen, China. Participants received a 12-month mHealth-HDTEP program delivered through the Shekangtong WeChat mini program, integrating blood pressure self-monitoring, health education, family doctor follow-up, and specialist consultation pathways. The primary outcome was HRQoL measured using the EQ-5D-5L, while secondary outcomes included systolic blood pressure (SBP) and diastolic blood pressure (DBP). Continuous pre-post changes were assessed using paired t-tests when normality assumptions were satisfied, and changes in EQ-5D-5L dimension levels were assessed using Wilcoxon signed-rank tests with false discovery rate (FDR) correction for multiple comparisons. Pearson correlation analyses were used to examine associations between changes in blood pressure and HRQoL measures.

**Results:**

Among 225 enrolled participants, 210 completed the 12-month follow-up. Compared with baseline, statistically significant improvements were observed in both blood pressure and HRQoL outcomes. Mean SBP decreased from 145.35 ± 4.18 mmHg to 130.64 ± 5.62 mmHg (mean difference: 14.71 mmHg, 95% *CI*: 13.68 to 15.74; *p* < 0.001; *Cohen’s d* = 2.043), and mean DBP decreased from 68.93 ± 4.85 mmHg to 60.73 ± 4.31 mmHg (mean difference: 8.20 mmHg, 95% *CI*: 7.28 to 9.12; *p* < 0.001; *Cohen’s d* = 1.262). EQ-5D-5L utility values increased from 0.82 ± 0.13 to 0.94 ± 0.06 (mean difference, 0.12; 95% *CI*, 0.10 to 0.14; *p* < 0.001; Cohen’s *d* = 0.850), and EQ-VAS scores increased from 74.16 ± 11.07 to 80.93 ± 8.03 (mean difference, 6.77; 95% *CI*, 6.18 to 7.36; *p* < 0.001; *Cohen’s d* = 1.536). The anxiety/depression dimension showed the most pronounced improvement among the five EQ-5D-5L dimensions (FDR-adjusted *p* < 0.001). Changes in blood pressure indicators were positively associated with changes in HRQoL measures (*p* < 0.001). Reductions in blood pressure were positively correlated with improvements in HRQoL parameters (*r* = 0.22 to 0.32, all *p* < 0.001).

**Conclusion:**

A mHealth-enabled hierarchical care model integrated within regional healthcare networks was associated with improvements in blood pressure indicators and HRQoL among older adults with hypertension. The findings suggest that combining digital self-management support with primary care coordination may represent a feasible strategy for supporting chronic disease management. Further controlled studies are required to determine effectiveness, sustainability, and scalability across diverse healthcare settings.

## 1 Introduction

Hypertension represents a major global public health challenge and ranks among the leading contributors to disability-adjusted life years (DALYs) worldwide (1). As a major risk factor for non-communicable diseases (NCDs), including cardiovascular disease, stroke, and renal failure, hypertension continues to impose substantial health and socioeconomic burdens globally (2–4). The rapid growth of the ageing population has further intensified this challenge, as older adults experience increasing susceptibility to multimorbidity and chronic disease progression (5). The accumulation of age-related health risks substantially affects health-related quality of life (HRQoL) among older populations (6). China faces particularly prominent challenges associated with population ageing (7). By 2030, the population aged 65 years and older in China is projected to reach 258 million, accounting for 18.23% of the total population (8). Meanwhile, approximately half of individuals aged 69 years and older in China are affected by hypertension, representing one of the largest hypertension burdens worldwide (9, 10). Despite continuous improvements in healthcare delivery, blood pressure control rates in China remain suboptimal compared with those in developed countries (11, 12).

The management of hypertension among older adults remains challenging due to the need for continuous monitoring, long-term behavioural modification, and sustained engagement with healthcare services (13). Traditional healthcare models that rely primarily on periodic face-to-face consultations are often insufficient for providing continuous disease management and timely health guidance (14). Rapid socioeconomic development, population ageing, and limited coordination between different levels of healthcare institutions have further increased pressure on hospital-centred healthcare systems (15). In particular, conventional outpatient-based management may limit opportunities for regular blood pressure monitoring and comprehensive health education, potentially affecting treatment adherence and self-management behaviours (16). Therefore, innovative approaches that support continuous hypertension management among older adults are urgently needed (17). Effective self-management is a key component of hypertension control, and structured interventions focusing on dietary modification, physical activity, medication adherence, and behavioural change have demonstrated benefits for chronic disease management (18). However, limited consultation time and insufficient health literacy may restrict the ability of healthcare providers to deliver sustained self-management support, leaving many patients with inadequate knowledge and skills for long-term disease management (19).

To address these challenges, China has extensively promoted the hierarchical diagnosis and treatment system (HDTS) nationwide as a central healthcare reform strategy. This system is organized around the principle of “minor illnesses managed in community settings, major illnesses treated in hospitals, and rehabilitation returned to communities” (20). For chronic conditions such as hypertension, this framework is widely implemented across the country, prioritizing long-term management at the primary healthcare level—including standardized blood pressure monitoring, health education, and regular follow-up by family doctor teams (21–23). To facilitate cross-tier collaboration, Regional Medical Consortia (RMCs) have been broadly established to vertically integrate tertiary hospitals with primary community healthcare centers, establishing pathways for two-way referrals, resource sharing, and specialist technical support (23, 24).

Despite this widespread policy rollout, routine implementation remains variable across regions; coordination between healthcare tiers is often limited by fragmented health data, delayed inter-institutional communication, and episodic, passive outpatient encounters. The present mHealth-enabled hierarchical care model (mHealth-HDTEP) directly extends and enhances existing pathways by embedding mobile internet and cloud-based data-sharing into the RMC infrastructure. Unlike traditional fragmented models, this digital platform enables real-time home blood pressure telemonitoring, automated threshold-based risk alerts, and seamless multidisciplinary communication between community doctors and tertiary specialists, thereby transforming intermittent clinical visits into a proactive, continuous, and closed-loop care pathway.

Similar digitally supported, integrated hypertension management models—combining remote patient monitoring with coordinated multidisciplinary support across primary and specialty care—have increasingly been investigated and implemented in other healthcare systems, such as the National Health Service (NHS) in the United Kingdom and integrated delivery networks in the United States (25, 26). However, care governance, provider responsibilities, and the degree of technology integration within routine public healthcare pathways vary considerably across countries. Contextualizing this mHealth-enabled hierarchical care model within China’s regional medical networks provides valuable insights for international health systems seeking scalable, digitally supported chronic disease management strategies.

Meanwhile, advances in mobile internet technologies and big data analytics have created new opportunities for strengthening chronic disease management through digital health approaches (27, 28). Previous studies have demonstrated that mHealth interventions may enhance patient engagement, health literacy, and self-management capabilities among individuals with chronic diseases (29, 30). Recent evidence suggests, however, that the effectiveness of digitally supported hypertension management depends not only on the technology itself but also on the way digital tools are integrated with ongoing healthcare support. A 2026 systematic review focusing specifically on older adults found that eHealth interventions incorporating active healthcare professional involvement and multiple components, such as lifestyle education, risk-factor management, and medication support, showed greater potential for improving blood pressure control than technology-based approaches alone. The review also highlighted that digital health effectiveness may vary according to digital health literacy, functional limitations, and access to digital technologies (31). Consistent with this evidence, systematic reviews of nurse-led and other professional-supported digital interventions have reported improvements in blood pressure control and self-management, suggesting that education, regular follow-up, feedback, and professional engagement may be important components of digitally supported hypertension care (32).

Importantly, the outcomes of hypertension management extend beyond blood pressure reduction. Recent evidence suggests that mobile health interventions may also improve treatment adherence, disease-related knowledge, self-management, and quality of life, highlighting the importance of patient-centred outcomes in evaluating digital hypertension care (33). HRQoL is particularly relevant among older adults because hypertension commonly occurs in the context of multimorbidity, functional limitations, and other age-related health challenges. Nevertheless, much of the existing literature has focused on blood pressure and behavioural outcomes, whereas the integration of sustained professional support, longitudinal digital monitoring, primary care coordination, and patient-reported HRQoL outcomes within routine healthcare systems remains less well characterized.

The present mHealth-enabled hierarchical care model builds on this existing organizational pathway rather than replacing it with a standalone digital intervention. The Shekangtong platform provides an additional digital layer that connects patient self-monitoring and health education with family doctor follow-up and specialist consultation within the RMC structure. In this way, patient-generated information can be incorporated into longitudinal primary care management while providing a pathway for additional specialist support when required. The distinguishing feature of the present model is therefore its integration of these digital functions into the existing hierarchical care and RMC framework in China. This prospective study aimed to evaluate longitudinal changes in blood pressure control and HRQoL associated with a 12-month mHealth-enabled hierarchical care model (mHealth-HDTEP) among older hypertensive adults.

## 2 Methods

### 2.1 Study design

This was a prospective, single-arm, pre-post evaluation study conducted from January 2025 to December 2025. The study evaluated changes in blood pressure and health-related quality of life from baseline to the 12-month follow-up after implementation of a mHealth-enabled hierarchical care model. Because the study did not include a concurrent control group, observed changes were interpreted as pre-post associations and were not considered evidence of a causal intervention effect. The study evaluated a mHealth-enabled hierarchical care model for hypertension management among older adults within regional medical consortia (RMCs) in Shenzhen, China. The study protocol was developed before study initiation, and reporting followed the Transparent Reporting of Evaluations with Nonrandomized Designs (TREND) guidelines.

### 2.2 Sample size calculation

The sample size was estimated based on the primary outcome, the EQ-5D-5L utility score, using a paired mean-difference framework (34):


$$N={\left(Z\_(\alpha /2)+Z\_\beta \right)}^{2}\cdot \sigma^{2}/\delta^{2}$$

Where (σ) denotes the anticipated standard deviation of the within-participant change in the EQ-5D-5L utility score. A two-sided significance level of 0.05 (Z_(α/2), 80%) statistical power (Z_β = 0.84), an anticipated standard deviation of the paired change of 0.25, and a prespecified clinically meaningful difference of 0.05 were assumed. Based on these parameters, the required minimum sample size was 196 participants. Allowing for approximately 10% attrition increased the required sample size to 218 participants. We therefore targeted recruitment of approximately 225 participants to provide an additional margin for loss to follow-up and variability across participating healthcare settings.

Shenzhen has established multiple RMCs integrating tertiary hospitals and primary healthcare institutions. In this study, participating RMCs were selected purposively to represent different geographic and healthcare settings. Eligible participants were recruited through collaborating healthcare institutions within these RMCs.

### 2.3 Participants

Between January 1 and December 30, 2025, a total of 225 older adults with primary hypertension were recruited from community health service centers affiliated with regional medical consortia (RMCs) in Shenzhen, China. In this study, the participant age criterion was prespecified as 60–70 years. Adults older than 70 years were excluded *a priori* to ensure a relatively homogeneous study population regarding baseline digital health literacy, multimorbidity burden, functional independence, and support requirements, all of which substantially influence user engagement and intervention adherence in digital health trials (35). The potential limitation regarding the generalizability of our findings to the broader population of adults older than 70 years is explicitly acknowledged in the Discussion section. The specific eligibility criteria for this study were as follows:

#### 2.3.1 Inclusion criteria

(1) age 60–70 years and diagnosis of hypertension according to the 2023 Chinese Hypertension Prevention and Treatment Guidelines (systolic blood pressure ≥140 mmHg and/or diastolic blood pressure ≥ 90 mmHg), with a disease duration of at least 1 year; (2) willingness to participate and ability to complete baseline and follow-up assessments; (3) basic ability to use smartphone-based services required for participation in the mHealth program; and (4) absence of severe target organ complications, including major cardiac, cerebral, or renal complications, or psychiatric disorders.

Medication Documentation: Baseline antihypertensive medication regimens (drug classes, doses, and adherence patterns) were documented at baseline enrollment from patient reports and clinical records. However, longitudinal changes in antihypertensive medication use were not prospectively tracked during the 12-month follow-up period. Participants were not explicitly instructed to maintain stable medication regimens, nor were medication changes systematically collected through pharmacy records, patient diaries, or clinical follow-up visits. This represents a substantial limitation discussed below.

#### 2.3.2 Exclusion criteria

(1) severe comorbid conditions that could substantially influence HRQoL assessment, such as malignant tumours or severe liver cirrhosis; (2) previous participation in similar mHealth intervention studies.

### 2.4 Intervention

All participants received the mHealth-enabled hierarchical diagnosis and treatment education program (HDTEP) delivered through the Shekangtong WeChat mini program (Community Health Pass). The platform was embedded within the WeChat ecosystem and provided access to chronic disease management services without requiring independent application installation. The system supported communication among patients, primary healthcare providers, and tertiary hospital specialists, thereby facilitating coordinated care within RMCs (36). The intervention integrated resources from the RMCs to support coordinated care.

As illustrated in Figure 1, the conceptual framework of the intervention involved interactions among patients, community health centres, and tertiary hospitals. The intervention integrated digital self-management support with primary care follow-up and specialist consultation pathways. The Shekangtong platform was integrated with RMC electronic health record systems.

**Figure 1:**
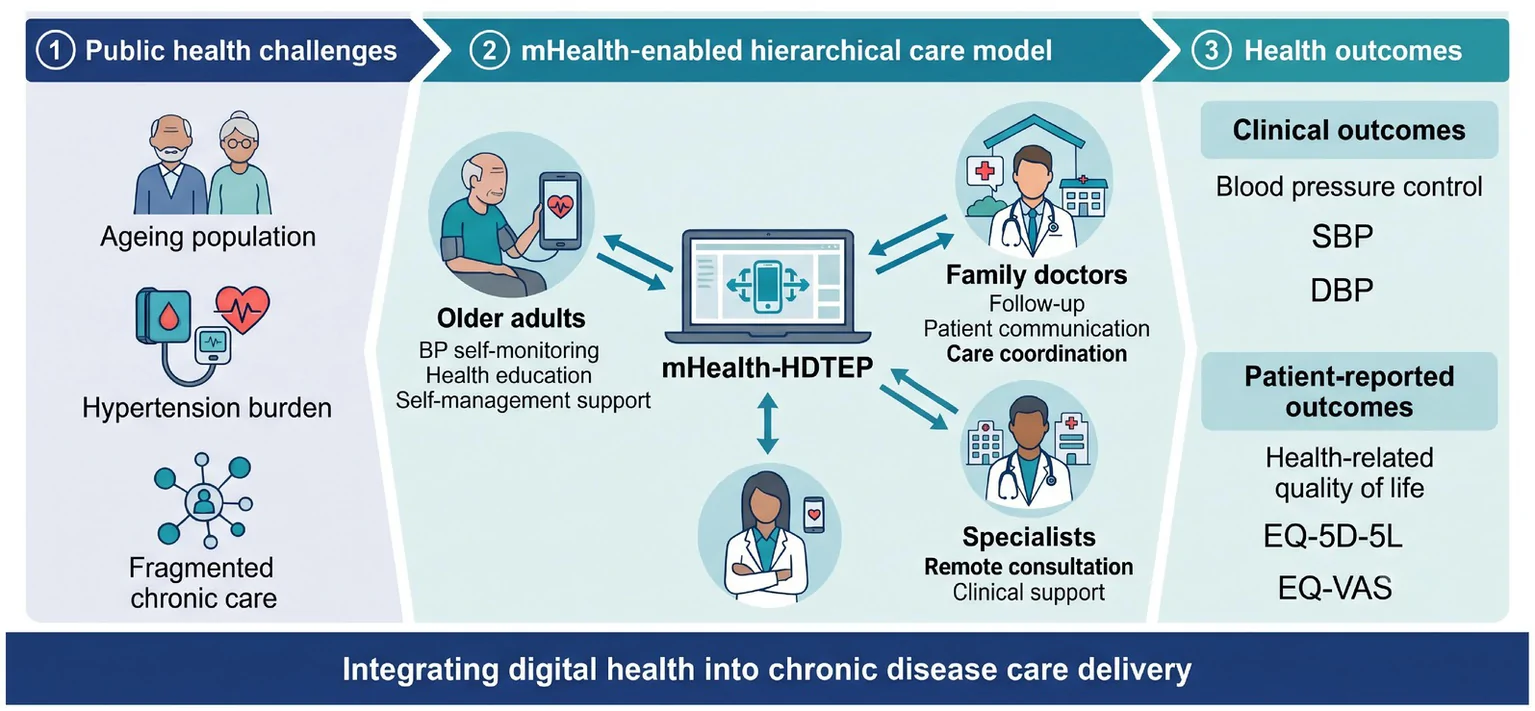
Conceptual framework of the mHealth-enabled hierarchical diagnosis and treatment education program (HDTEP).

The intervention comprised five components: (1) participants were encouraged to perform daily home blood pressure monitoring and record measurements through the Hypertension Manager, which maintained longitudinal blood pressure records and trends; (2) personalized health education content was delivered two to three times per week and covered hypertension prevention, dietary management, physical activity, and lifestyle modification; content was tailored according to participant characteristics, including age and education level; (3) participants could initiate remote consultations with family doctors through the “Consult Your GP” and “Book Your GP” functions, and family doctors responded to health-related inquiries within 24 h;(4)when additional clinical assessment or specialist support was considered necessary, family doctors communicated with tertiary hospital specialists through the RMC pathway; (5) monthly online health education and experience-sharing activities were provided.

No fixed frequency of one-to-one participant contacts beyond the documented platform-based consultation pathway was prespecified. The duration of individual educational deliveries and monthly online sessions was not prospectively recorded. Likewise, a formal checklist-based intervention fidelity assessment was not performed. Participant engagement was assessed using platform-recorded blood pressure uploads, with the average number of uploaded measurements calculated over the 12-month period.

### 2.5 Outcome measures and assessment tools

#### 2.5.1 Primary outcome measure

HRQoL was assessed using the EuroQol Five-Dimensional Five-Level questionnaire (EQ-5D-5L). The EQ-5D-5L is a standardized instrument for assessing health-related quality of life (HRQoL) and health status across five dimensions: mobility, self-care, usual activities, pain/discomfort, and anxiety/depression (37). The instrument was selected because of its concise format, ease of administration, and suitability for older populations (38). Each dimension contains five response levels ranging from no problems to extreme problems. Participants’ responses generate a five-digit health state profile, and utility scores were calculated using the Chinese value set (39). The EQ-VAS was also used to assess participants’ overall perceived health status, ranging from 0 (worst imaginable health) to 100 (best imaginable health). The Chinese version of the EQ-5D-5L has been translated and validated by the EuroQol Group, and previous studies have demonstrated its reliability and validity among Chinese patients with hypertension (40).

#### 2.5.2 Secondary outcome measures

Blood pressure was assessed at baseline and at the 12-month follow-up by trained research personnel using a calibrated electronic sphygmomanometer. Measurements were obtained in the morning after fasting and a period of rest. Three consecutive blood pressure readings were obtained at 5-min intervals, and the mean of the second and third readings was used to derive systolic blood pressure (SBP) and diastolic blood pressure (DBP) for analysis. The same measurement procedure was applied at both baseline and follow-up.

These endpoint measurements were obtained directly by trained research personnel and were not derived from participants’home-monitoring records. Participants were also encouraged to perform daily home blood pressure self-monitoring through the Hypertension Manager. Platform-recorded home blood pressure measurements were used to support longitudinal management but were not used as the endpoint blood pressure values. Participants were instructed and encouraged to perform home blood pressure (BP) monitoring daily, preferably in the morning (within 1 h after waking, after bladder emptying, and before medication or breakfast) and/or evening, using validated upper-arm electronic monitors. Platform engagement was quantified as the average number of weekly uploaded BP measurements per participant over the 12-month period. The platform maintained longitudinal blood pressure records and trends that could be accessed by the healthcare team to support ongoing management. The study did not prespecify a formal adherence threshold for home blood pressure monitoring or a standardized automated alert threshold for markedly elevated blood pressure. When participants required medical advice, they could contact their family doctors through the platform, and specialist consultation was arranged through the RMC pathway when additional clinical support was considered necessary.

### 2.6 Data quality control

Data collection and entry were performed by trained research personnel. Range checks and double-entry verification procedures were applied to improve data accuracy. Participant identifiers were replaced with unique study codes before analysis.

### 2.7 Statistical analysis

All statistical analyses were performed using R version 4.5.2. Data entry and validation were conducted using EpiData version 3.1 to ensure data quality and accuracy. Continuous variables were assessed for distributional characteristics before analysis. Normally distributed variables were summarized as mean ± standard deviation (SD), whereas non-normally distributed variables were presented as median and interquartile range (IQR). Categorical variables were summarized as frequencies and percentages. Descriptive analyses were conducted to characterize participants’ sociodemographic characteristics, clinical profiles, and healthcare-related factors. Participants who completed both baseline and follow-up assessments were included in the primary analysis, and missing outcome data were handled according to the predefined analysis plan.

Changes in continuous outcomes before and after the intervention, including EQ-5D-5L utility scores, EQ-VAS scores, systolic blood pressure (SBP), and diastolic blood pressure (DBP), were assessed using paired t-tests when normality assumptions were satisfied. Standardized effect sizes (Cohen’s *d*) were calculated to quantify the magnitude of pre-post changes. Changes in the five EQ-5D-5L dimension levels were assessed using the Wilcoxon signed-rank test for paired ordinal data, with false discovery rate (FDR) correction applied to account for multiple comparisons.

Pearson correlation analyses were performed to examine associations between changes in blood pressure indicators and changes in HRQoL measures when the normality assumption was satisfied. Correlation coefficients were interpreted as measures of association and not causal effects. All statistical tests were two-sided, and *p* < 0.05 was considered statistically significant.

## 3 Results

### 3.1 Participant characteristics and follow-up

A total of 225 participants with hypertension were enrolled in the study. Among them, 210 participants completed the 12-month follow-up, resulting in a retention rate of 93.3%. The primary analysis included the 210 participants who completed both baseline and follow-up assessments. No imputation was performed for missing outcome data, and the primary analysis was restricted to participants with complete baseline and follow-up assessments. Baseline characteristics were not formally compared between completers and non-completers due to the small sample size of those lost to follow-up (*n* = 15); however, loss to follow-up was distributed across relocation (*n* = 7), incapacitating comorbidities (*n* = 5), and voluntary withdrawal (*n* = 3), suggesting diverse rather than health-related reasons for discontinuation. The participant recruitment and follow-up process is illustrated in Figure 2.

**Figure 2:**
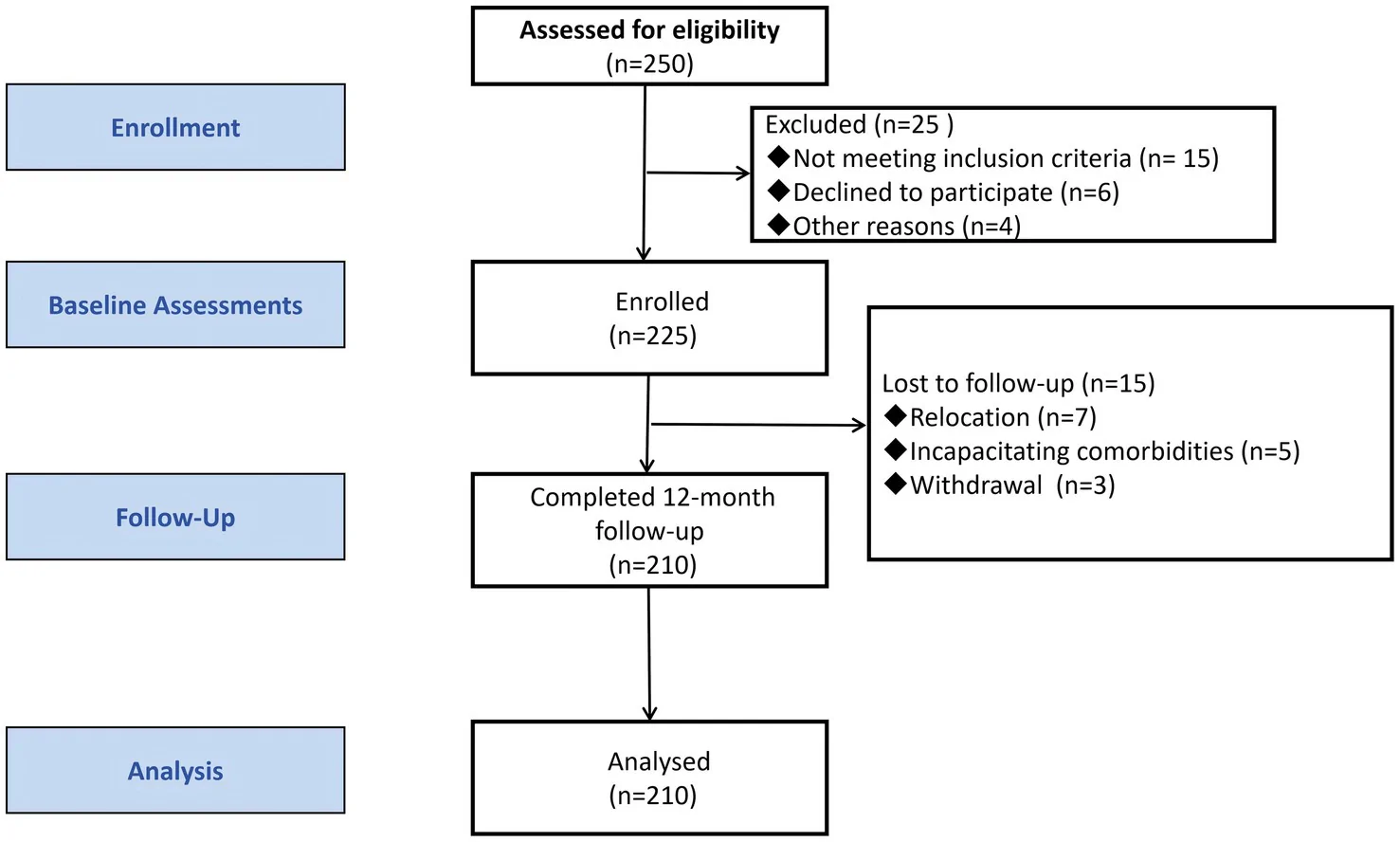
Flow diagram of participant recruitment, follow-up, and analysis.

Baseline characteristics of the 210 participants included in the final analysis are presented in Table 1. The mean age of participants was 63.32 ± 3.09 years, with 116 (55.2%) participants aged 60–65 years and 94 (44.8%) aged 66–70 years. Among participants, 114 (54.3%) were male and 96 (45.7%) were female. Participant engagement was assessed using platform-recorded blood pressure uploads. Engagement with the mHealth-enabled hierarchical care model was monitored throughout the intervention period. Participants uploaded blood pressure measurements an average of 3.2 ± 1.4 times per week through the digital platform. No intervention-related adverse events were reported during the study period. Although blood pressure uploads provided a measure of platform engagement, more comprehensive indicators of intervention exposure, adherence to other intervention components, and intervention fidelity were not systematically collected.

**Table 1:** Baseline sociodemographic, clinical, and healthcare-related characteristics of participants with hypertension (*N* = 210).

Characteristic	Category	*N* (%)
Sociodemographic		
Sex	Male	114 (54.3)
	Female	96 (45.7)
Age, years	60–65	116 (55.2)
	66–70	94 (44.8)
Education	Primary school and below	70 (33.3)
	Junior high school	36 (17.1)
	Senior high or technical school	51 (24.3)
	College or above	53 (25.2)
Marital status	Married	155 (73.8)
	Single	47 (22.4)
	Widowed	8 (3.8)
Monthly income(RMB^a^)	≤5,000	59 (28.1)
	5,001–8,000	57 (27.1)
	8,001–11,000	47 (22.4)
	≥11,001	47 (22.4)
Main sources of income	Retirement allowance	60(28.6)
	Child support	43(20.5)
	Income from own labor	62(29.5)
	Social support	45(21.4)
Living status	With spouse	84(40.0)
	With spouse and children	54(25.7)
	With a caregiver	36(17.1)
	With children	27(12.9)
	Living alone	9(4.3)
Clinical characteristics		
Duration of hypertension,years	1–5	91 (43.3)
	6–10	85 (40.5)
	11–15	25 (11.9)
	15–20	9 (4.3)
Comorbidities	Yes	154 (73.3)
	None	56 (26.7)
BP^b^ monitoring frequency	Once a day	27 (12.9)
	Once a week	82 (39.0)
	Once a month	82 (39.0)
	Never	19 (9.0)
Health system factors		
Funding for medical expenses	Self paying	80 (38.1)
	Urban and rural medical insurance	70 (33.3)
	Employee medical insurance	40 (19.1)
	Commercial health insurance	20 (9.5)
BP control strategy	Medication	125 (59.5)
	Change eating habits	66 (31.4)
	Increase physical activity	10 (4.8)
	Reduces stress and anxiety	4 (1.9)
	Other strategies (eg, smoking cessation)	5 (2.4)
Sufficient healthcare professional support	No	157 (74.8)
	Yes	53 (25.2)

^a^ RMB, Renminbi (currency of China).

^b^ BP, blood pressure.

Regarding socioeconomic characteristics, 104 participants (49.5%) had completed senior high school or higher education. Most participants lived with relatives or caregivers, with 165 (78.6%) participants reporting family-based living arrangements. Thirty-six participants (17.1%) lived with caregivers, while 9 (4.3%) lived alone.

Regarding clinical characteristics, the mean duration of hypertension was 7.17 ± 5.11 years, and 154 participants (73.3%) reported at least one comorbidity. Baseline hypertension self-management behaviours indicated limited regular blood pressure monitoring, with only 27 participants (12.9%) measuring blood pressure daily, whereas 19 participants (9.0%) reported never monitoring blood pressure.

In terms of healthcare-related characteristics, medication use was the most common strategy for blood pressure control (125/210, 59.5%), followed by dietary modification (66/210, 31.4%). A substantial proportion of participants (157/210, 74.8%) reported insufficient professional healthcare support before the intervention. Regarding medical expenditure coverage, 130 participants (61.8%) received financial support through health insurance schemes, whereas 80 participants (38.1%) reported self-payment.

### 3.2 Changes in EQ-5D-5L dimensions

Changes in the five EQ-5D-5L dimensions before and after the intervention are presented in Table 2. Significant changes were observed in several HRQoL dimensions between baseline and the 12-month follow-up.

**Table 2:** Comparison of the distribution of EQ-5D-5L dimension levels among participants before and after the mHealth-HDTEP intervention (*N* = 210).

Dimension and level	Pre-intervention, *N* (%)	Post-intervention, *N* (%)	*p*-value^a^	FDR adjusted *p*-value^b^
Mobility			0.007	0.0194
No problems	146 (69.5)	162 (77.1)		
Slight problems	55 (26.2)	48 (22.9)		
Moderate problems	9 (4.3)	0 (0.0)		
Severe or extreme problems	0 (0.0)	0 (0.0)		
Self-care			0.13	0.167
No problems	169 (80.5)	176 (83.8)		
Slight problems	36 (17.1)	34 (16.2)		
Moderate problems	5 (2.4)	0 (0.0)		
Severe or extreme problems	0 (0.0)	0 (0.0)		
Usual activities			0.33	0.329
No problems	150 (71.4)	157 (74.8)		
Slight problems	55 (26.2)	50 (23.8)		
Moderate problems	5 (2.4)	3 (1.4)		
Severe or extreme problems	0 (0.0)	0 (0.0)		
Pain/discomfort			0.05	0.0877
No problems	141 (67.1)	155 (73.8)		
Slight problems	64 (30.5)	55 (26.2)		
Moderate problems	5 (2.4)	0 (0.0)		
Severe or extreme problems	0 (0.0)	0 (0.0)		
Anxiety/depression			<0.001	<0.001
No problems	68 (32.4)	178 (84.8)		
Slight problems	87 (41.4)	32 (15.2)		
Moderate problems	55 (26.2)	0 (0.0)		
Severe or extreme problems	0 (0.0)	0 (0.0)		

^a^ P values were calculated using the Wilcoxon signed-rank test.

^b^ FDR, False discovery rate.

The largest change was observed in the anxiety/depression dimension. At baseline, 68 participants (32.4%) reported no problems in this dimension, whereas this proportion increased to 178 participants (84.8%) at follow-up (FDR adjusted *p* < 0.001). In addition, the proportion of participants reporting moderate problems decreased from 55 (26.2%) at baseline to 0 (0.0%) at follow-up.

Important interpretation note: This substantial improvement in the anxiety/depression dimension should NOT be interpreted as evidence that the intervention treats clinical anxiety disorder or major depression. The EQ-5D-5L anxiety/depression item measures perceived health-related distress associated with one’s overall health status, not formal psychiatric diagnosis. The observed improvement likely reflects: (1) reduced health-related anxiety secondary to improved blood pressure control and disease confidence; (2) increased sense of clinical support and engagement with family doctors; (3) improved general well-being accompanying better hypertension management. Assessment of whether this mHealth model affects clinically diagnosed anxiety or depression would require validated psychiatric instruments such as the Patient Health Questionnaire-9 (PHQ-9) or Generalized Anxiety Disorder-7 (GAD-7) scale. Clinicians and readers should not interpret these EQ-5D-5L findings as evidence of psychiatric treatment effects.

Changes were also observed in the mobility dimension. The proportion of participants reporting no mobility problems increased from 146 (69.5%) at baseline to 162 (77.1%) at follow-up (FDR adjusted *p* = 0.019), while the proportion reporting moderate problems decreased from 9 (4.3%) to none.

For the pain/discomfort dimension, the proportion of participants reporting no problems increased from 141 (67.1%) at baseline to 155 (73.8%) at follow-up; however, this change was not statistically significant after FDR correction (FDR adjusted *p* = 0.088).

Although favourable changes were observed in the self-care and usual activities dimensions, these differences did not reach statistical significance (FDR adjusted *p* = 0.167 and 0.329, respectively). No participants reported severe or extreme problems in any EQ-5D-5L dimension at either assessment point.

### 3.3 Changes in blood pressure outcomes

Changes in systolic and diastolic blood pressure before and after the intervention are summarized in Table 3. The mean systolic blood pressure decreased from 145.35 ± 4.18 mmHg at baseline to 130.64 ± 5.62 mmHg at the 12-month follow-up. The mean change was 14.71 ± 7.20 mmHg (95% *CI*, 13.68–15.74, *p* < 0.001).

**Table 3:** Changes in blood pressure measurements before and after the mHealth-enabled hierarchical care intervention (*N* = 210).

Variable	Baseline, mean ± SD	Follow-up, mean ± SD	Mean change, mean ± SD	95% CI	*p-* value^c^	Cohen’s *d*^d^
SBP (mmHg)^a^	145.35 ± 4.18	130.64 ± 5.62	14.71 ± 7.20	13.68 to 15.74	<0.001	2.043
DBP (mmHg)^b^	68.93 ± 4.85	60.73 ± 4.31	8.20 ± 6.50	7.28 to 9.12	<0.001	1.262

^a^ SBP, systolic blood pressure.

^b^ DBP, diastolic blood pressure.

^c^ *P* values were obtained using paired *t*-tests.

^d^ Cohen’s *d* was calculated as the mean paired change divided by the standard deviation of the paired differences.

Similarly, diastolic blood pressure decreased from 68.93 ± 4.85 mmHg at baseline to 60.73 ± 4.31 mmHg at follow-up, with a mean change of 8.20 ± 6.50 mmHg (95% *CI*, 7.28–9.12, *p* < 0.001).

These findings indicate that blood pressure indicators improved over the 12-month follow-up period. Given the single-arm pre-post design, these observed changes should not be interpreted as evidence of a causal effect of the mHealth-enabled hierarchical care model. Visual representations of these changes are presented in Figure 3.

**Figure 3:**
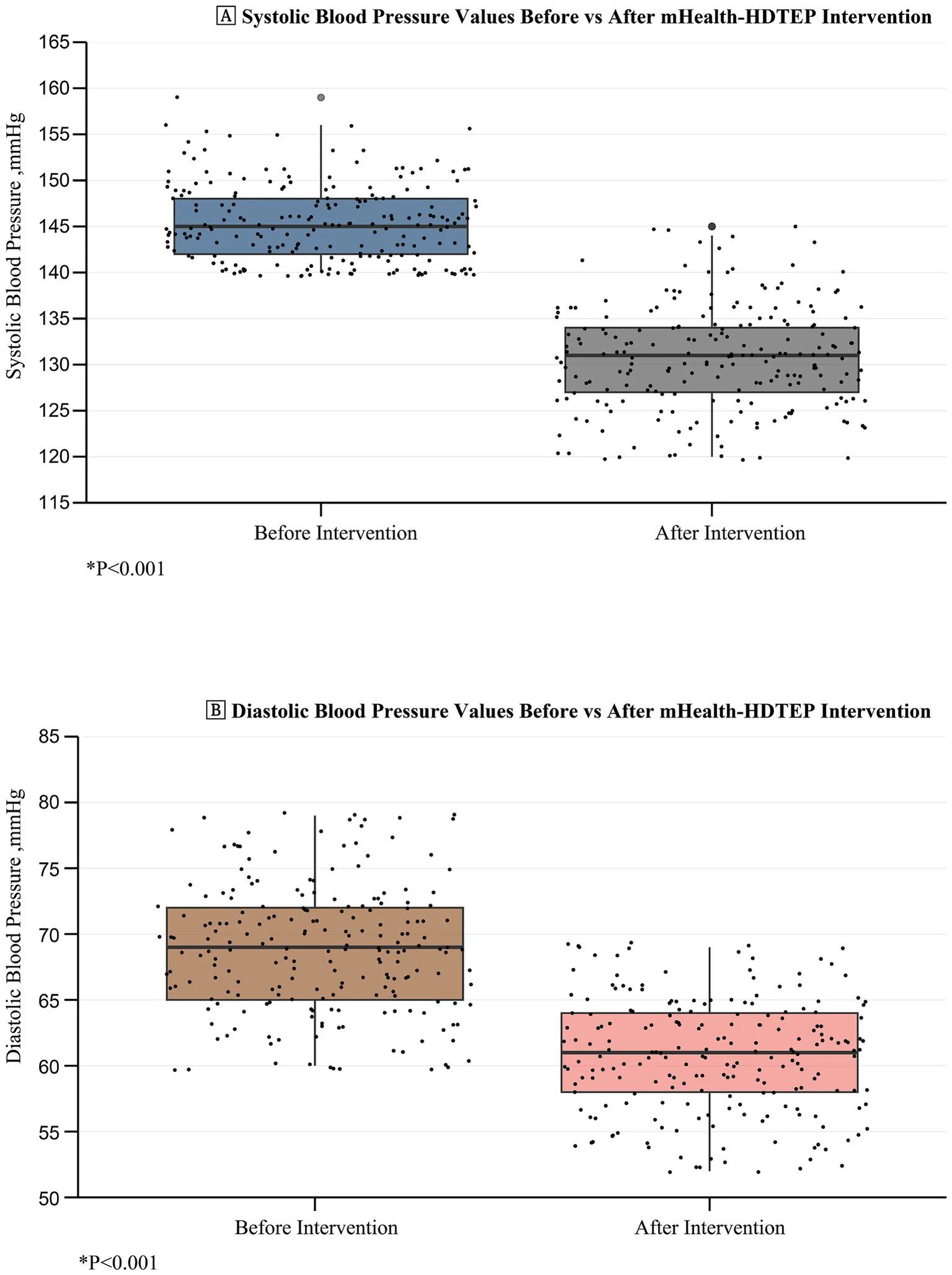
Changes in systolic and diastolic blood pressure from baseline to the 12-month follow-up. **(A)** Systolic blood pressure (SBP). **(B)** Diastolic blood pressure (DBP).

### 3.4 Primary outcome: health-related quality of life

Changes in HRQoL outcomes are presented in Table 4. Improvements in both EQ-5D-5L utility values and EQ-VAS scores were observed after the 12-month mHealth-enabled hierarchical care intervention.

**Table 4:** Changes in health-related quality of life outcomes before and after the mHealth-enabled hierarchical care intervention (*N* = 210).

Variable	Baseline, mean ± SD	Follow-up, mean ± SD	Mean change, mean ± SD	95% CI	*p-*value^c^	Cohen’s *d*^d^
EQ-5D-5L utility value^a^	0.82 ± 0.13	0.94 ± 0.06	0.12 ± 0.14	0.10 to 0.14	<0.001	0.850
EQ-VAS score^b^	74.16 ± 11.07	80.93 ± 8.03	6.77 ± 4.41	6.18 to 7.36	<0.001	1.536

^a^ EQ-5D-5L utility value: a summary index that reflects the overall health status, typically ranging from 0 (worst health) to 1 (perfect health).

^b^ EQ-VAS, EuroQol Visual Analogue Scale. A score measuring the respondent’s perceived health status, ranging from 0 (worst health imaginable) to 100 (best health imaginable).

^c^ *P* values were obtained using paired *t*-tests.

^d^ Cohen’s d was calculated as the mean paired change divided by the standard deviation of the paired differences.

The mean EQ-5D-5L utility value increased from 0.82 ± 0.13 at baseline to 0.94 ± 0.06 at follow-up. The mean difference was 0.12 ± 0.14 (95% *CI*, 0.10–0.14; *p* < 0.001).

Similarly, the mean EQ-VAS score increased from 74.16 ± 11.07 at baseline to 80.93 ± 8.03 after the intervention, with a mean change of 6.77 ± 4.41 points (95% *CI*, 6.18–7.36; *p* < 0.001).

The improvements observed in the overall HRQoL indicators were consistent with the domain-specific changes identified in the EQ-5D-5L dimensions (Table 2), particularly in anxiety/depression, mobility, and pain/discomfort domains. Visual representations of these trends are provided in Figure 4.

**Figure 4:**
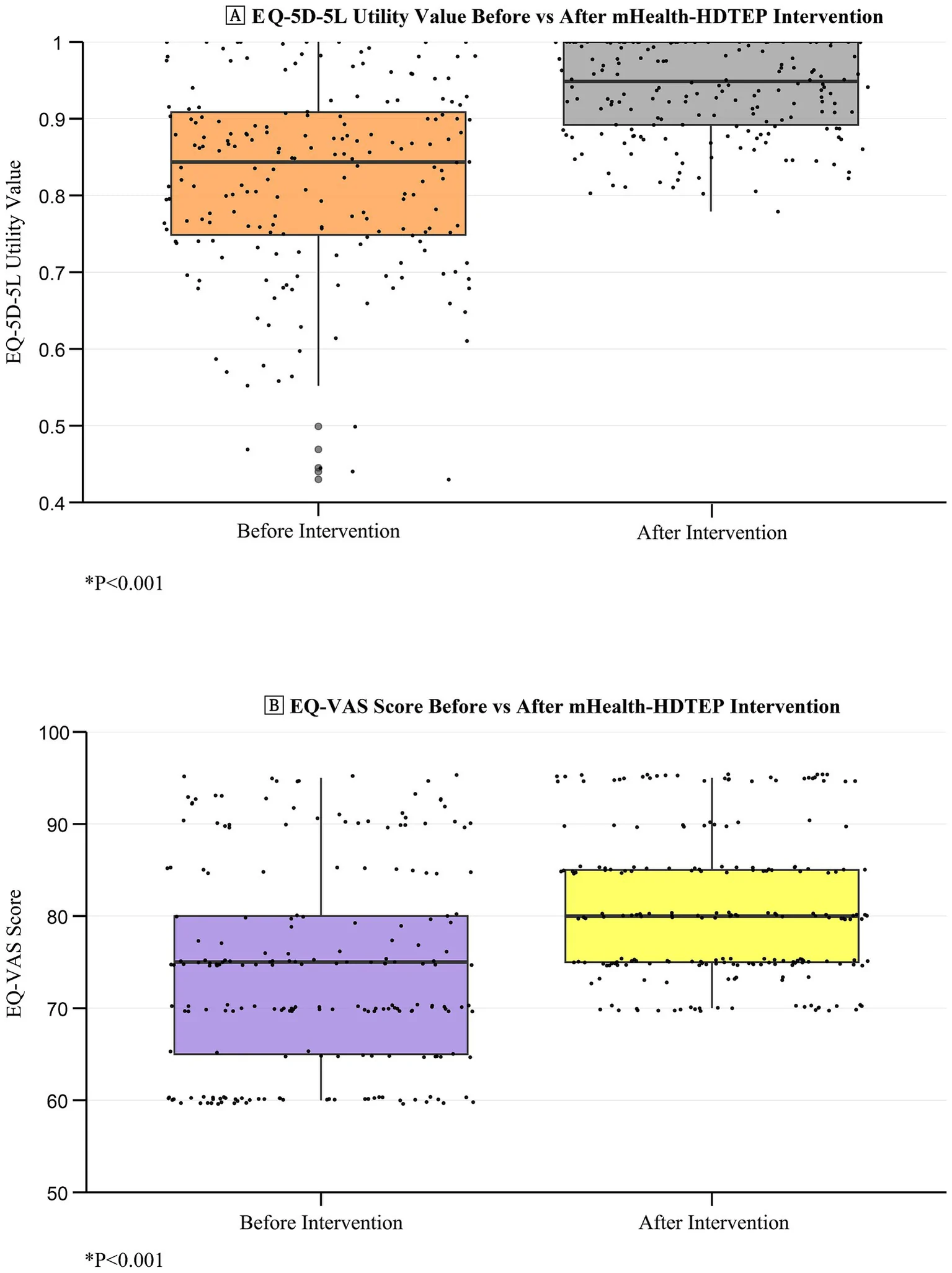
Changes in health-related quality of life from baseline to the 12-month follow-up. **(A)** Changes in EQ-5D-5L utility scores. **(B)** Changes in EQ-VAS scores. Change values were calculated as follow-up minus baseline.

### 3.5 Association between changes in HRQoL and blood pressure indicators

Pearson correlation analyses were performed to explore the associations between changes in blood pressure indicators and changes in HRQoL outcomes (Table 5; Figure 5).

**Table 5:** Correlations between changes in blood pressure indicators and changes in HRQoL outcomes (*N* = 210).

Variables	Correlation coefficient (r)	95% CI	*p-*value
EQ-5D-5L utility value^a^ vs. SBP^b^	0.32	0.21 to 0.43	<0.001
EQ-5D-5L utility value vs. DBP^c^	0.28	0.17 to 0.39	<0.001
EQ-VAS^d^ score vs. SBP	0.25	0.14 to 0.36	<0.001
EQ-VAS score vs. DBP	0.22	0.11 to 0.33	<0.001

^a^ EQ-5D-5L utility value: a summary index that reflects the overall health status, typically ranging from 0 (worst health) to 1 (perfect health).

^b^ SBP, systolic blood pressure.

^c^ DBP, diastolic blood pressure.

^d^ EQ-VAS, EuroQol Visual Analogue Scale. A score measuring the respondent’s perceived health status, ranging from 0 (worst health imaginable) to 100 (best health imaginable).

**Figure 5:**
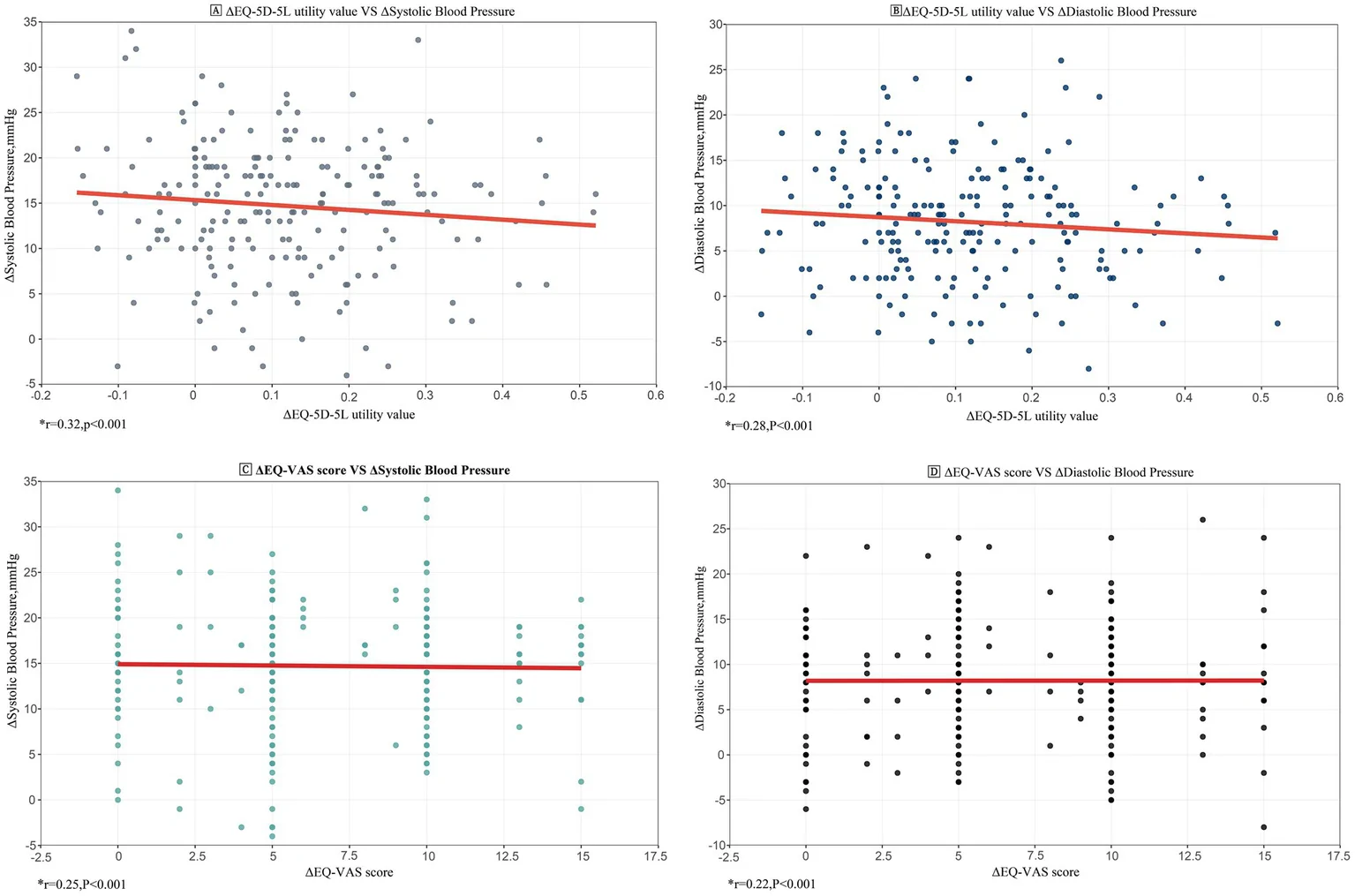
Associations between changes in health-related quality of life (HRQoL) measures and blood pressure from baseline to the 12-month follow-up. **(A)** ΔEQ-5D-5L utility score versus ΔSBP. **(B)** ΔEQ-5D-5L utility score versus ΔDBP. **(C)** ΔEQ-VAS score versus ΔSBP. **(D)** ΔEQ-VAS score versus ΔDBP. ΔEQ-5D-5L and ΔEQ-VAS were calculated as follow-up minus baseline values, whereas ΔSBP and ΔDBP were calculated as baseline minus follow-up values.

Reductions in systolic blood pressure were positively correlated with improvements in EQ-5D-5L utility values (*r* = 0.32, 95% *CI*, 0.21–0.43; *p* < 0.001) and EQ-VAS scores (*r* = 0.25, 95% *CI*, 0.14–0.36; *p* < 0.001).

Similarly, reductions in diastolic blood pressure were associated with increases in EQ-5D-5L utility values (*r* = 0.28, 95% *CI*, 0.17–0.39; *p* < 0.001) and EQ-VAS scores (*r* = 0.22, 95% *CI*, 0.11–0.33; *p* < 0.001).

These findings suggest that greater improvements in blood pressure indicators were accompanied by greater improvements in self-reported health status. However, given the observational pre-post study design, these correlations should be interpreted as associations rather than causal relationships.

## 4 Discussion

### 4.1 Principal findings

Critical Note on Study Design and Causal Inference: This prospective pre-post evaluation study without a concurrent control group cannot establish causation. The observed improvements in blood pressure and health-related quality of life represent pre-post associations that may have been influenced by multiple non-intervention factors, including: (1) regression to the mean (participants with elevated baseline values tend to show reduction on retesting); (2) natural disease course; (3) secular trends in healthcare practice; (4) Hawthorne effects (behavior change from study participation itself); (5) concurrent lifestyle modifications unrelated to the intervention; (6) unmeasured medication changes (addressed in detail below); and (7) other unmeasured clinical events or healthcare interactions. Readers should interpret all findings as preliminary evidence of associations warranting further controlled investigation, rather than as definitive proof of intervention effectiveness. Randomized controlled trials with concurrent control groups are essential to determine the true independent effect of the mHealth-enabled hierarchical care model.

This prospective pre–post study evaluated a mobile health–enabled hierarchical care model (mHealth-HDTEP) integrated into regional medical consortia among adults aged 60–70 years with hypertension. Over 12 months, participants demonstrated clinically meaningful reductions in systolic and diastolic blood pressure, accompanied by concurrent improvements in health-related quality of life and visual analogue scale scores. The proportion of participants reporting no problems in the anxiety/depression dimension also increased substantially. These outcomes occurred in the context of sustained platform engagement, structured home blood pressure monitoring, routine communication with primary care doctors, and streamlined access to specialist consultations.

The overall direction of these findings is consistent with recent evidence that mHealth interventions can improve blood pressure control and self-management among middle-aged and older adults with hypertension. A systematic review and meta-analysis of 14 randomized controlled trials involving 6,292 participants found that mHealth interventions improved systolic and diastolic blood pressure, blood pressure control, medication adherence, hypertension knowledge, and quality of life (33). Evidence from broader telehealth research has similarly demonstrated modest but sustained improvements in blood pressure across follow-up periods (41). Nevertheless, these findings reflect longitudinal observational associations rather than definitive causal effects. In the absence of a parallel control group, non-specific drivers—such as regression to the mean, secular trends, concurrent lifestyle adaptations, and unmeasured healthcare interactions—cannot be excluded.

### 4.2 Blood pressure changes

The observed reductions of 14.71 mmHg in systolic and 8.20 mmHg in diastolic blood pressure represent substantial absolute clinical improvements. Notably, these within-person declines exceed the pooled between-group differences typically documented in controlled telemonitoring literature. For example, a meta-analysis of 50 mHealth trials reported pooled between-group reductions of approximately 3.5 mmHg in systolic and 1.8 mmHg in diastolic blood pressure compared with usual care, though greater reductions emerged in selected high-risk or older cohorts (42). Similarly, a 12-month trial among older adults with dual diagnoses of hypertension and diabetes noted a 6.9 mmHg between-group difference (30).

Important Consideration—Medication Confounding: The substantial SBP reduction observed in this study (14.71 mmHg) warrants careful interpretation in light of a critical limitation: longitudinal medication changes were not systematically tracked. Given that: (1) our observed reduction magnitude aligns with standard antihypertensive medication effects; (2) baseline SBP of 145.35 mmHg indicated suboptimal control warranting clinical intensification; and (3) family doctors had routine contact with participants through the mHealth platform, medication intensification during the study period is probable. We cannot determine the independent contribution of the mHealth intervention versus concurrent medication changes. This distinction is essential: if the digital support primarily operated through improved medication-taking or clinical intensification, the mechanism differs substantially from effects through behavioral change or self-management alone. We therefore interpret the observed blood pressure improvements as pre-post associations occurring within a context of integrated care, rather than as direct intervention effects. Prospective medication tracking in future studies will be essential to disentangle these mechanisms.

The mHealth-HDTEP model incorporated repeated self-monitoring, personalized health education, family doctor follow-up, and specialist referral pathways, which may represent plausible pathways linking digital support with longitudinal hypertension management. However, the present study did not directly measure medication adherence, behavioural adherence, clinical response time, or other intermediate mechanisms. These proposed pathways should therefore be considered hypotheses for future evaluation rather than demonstrated mechanisms of the observed blood pressure changes.

### 4.3 Health-related quality of life

Generic health-related quality of life also showed marked improvements over the intervention period. The EQ-5D-5L utility index increased by an average of 0.12, and the EQ-VAS improved by nearly 7 points. These improvements echo recent syntheses demonstrating that digital health solutions promote broader well-being alongside clinical biomarker control (43). In older populations specifically, a 2026 meta-analysis confirmed that digital health interventions foster measurable improvements in physical functioning, general health perceptions, and psychological dimensions (44).

The largest improvement among the five EQ-5D-5L dimensions was in the anxiety/depression domain, with the proportion reporting no anxiety/depression problems increasing from 32.4% at baseline to 84.8% at follow-up. This substantial change (52.4 percentage point increase) warrants careful interpretation. First, this improvement should be understood as a change in perceived health-related distress rather than treatment or remission of clinically diagnosed anxiety or depressive disorders. The EQ-5D-5L anxiety/depression item is designed to capture general health related emotional distress across the population, not to diagnose or measure severity of specific psychiatric conditions. This distinction is important for clinical and policy audiences who might otherwise interpret the findings as evidence for psychiatric benefits of mHealth hypertension management. Second, the observed improvement likely reflects multiple mechanisms: (1) diseaserelated anxiety reduction—improved blood pressure control and confidence in disease management reduce hypertension-related worry; (2) care engagement effects—regular contact with family doctors through the platform may increase perceived clinical support and sense of partnership in care; (3) general health perception improvements—better symptom control and quality-of-life measures often co-occur with reduced health-related emotional distress. Third, these findings do not establish that this intervention is effective for treating clinical anxiety disorders or depression. To make such claims would require: (a) use of validated psychiatric instruments (PHQ-9, GAD-7, or clinical diagnostic interviews); (b) assessment of participants with baseline diagnosed anxiety/depression; and (c) comparison of psychiatric outcomes between intervention and control groups. These EQ-5D-5L findings are noteworthy for suggesting that integrated hypertension management may improve broader health-related well-being dimensions, but should not be misrepresented as psychiatric interventions. Future research incorporating validated mental health assessment tools would illuminate whether improved cardiometabolic outcomes and perceived healthcare engagement translate to reduced clinically significant anxiety or depression symptoms.

### 4.4 Integration of mHealth with hierarchical care

A defining characteristic of mHealth-HDTEP was its structural embedding within an operational hierarchical healthcare delivery system, avoiding the pitfalls of fragmented, stand-alone mobile applications. The digital platform served as an operational bridge: participants entered self-monitoring metrics and accessed educational materials, family doctors provided continuous frontline management, and tertiary specialists delivered consultation or coordinated upward transfers when indicated. This tiered integration appears exceptionally suited for older adults, whose engagement with digital health is frequently challenged by variable digital literacy, cognitive reserves, or limited informal caregiver support (45).

The primary value of this architecture lies in translating passive patient-generated health data into timely, coordinated clinical decisions. The digital platform was designed to connect patient-generated health information with primary and specialist care. In practice, family doctors provided routine follow-up and could seek specialist input when additional clinical assessment was required. However, the present study did not directly evaluate the effects of this architecture on clinician workload, response time, referral volume, or patient safety.

### 4.5 Limitations

We acknowledge the following limitations to this evaluation.

1. The single-arm pre–post design without a concurrent control group limits causal inference. Although reductions in blood pressure and improvements in HRQoL were observed over 12 months, these findings should be interpreted as associations rather than causal effects, as they may have been influenced by the natural course of hypertension, regression to the mean, participation-related changes in health awareness, including possible Hawthorne effects, concurrent lifestyle changes, and other unmeasured factors. Randomized controlled trials with appropriate concurrent control groups are therefore needed to determine the independent effect of the intervention.
2. Antihypertensive Medication Changes as Unmeasured Confounder: A critical limitation of this study is the absence of systematic tracking of longitudinal changes in antihypertensive medication use. Participants were not prospectively monitored for medication initiation, dose adjustment, or discontinuation over the 12-month period. This represents a substantial source of unmeasured confounding for several reasons: First, the magnitude of the observed SBP reduction (14.71 mmHg) aligns closely with the typical effects of antihypertensive medications in clinical trials (10–15 mmHg) and meta-analyses. Without medication data, we cannot determine what proportion of the observed reduction resulted from: (a) the digital intervention itself; (b) medication intensification; (c) improved medication adherence; or (d) regression to the mean combined with medication changes. Second, in the clinical context of this study, medication intensification is highly likely. At baseline, 59.5% of participants relied primarily on medication for blood pressure control, yet mean baseline SBP (145.35 mmHg) indicated inadequate control. During a 12- month intervention that emphasized structured follow-up and disease education, family doctors would reasonably intensify antihypertensive therapy in participants with persistent elevation. The absence of data on these medication changes prevents us from evaluating whether observed improvements stemmed from the mHealth platform or from concurrent clinical decision-making. Third, medication adherence changes—potentially improved through health literacy gains or repeated contact—could independently reduce blood pressure without attributing effects to digital monitoring or care coordination. To address this limitation, future studies should: (a) prospectively document all medication changes with dates and dosing information; (b) use pharmacy claims data when available; (c) employ medication adherence measurement tools (e.g., Morisky Scale); and (d) conduct sensitivity analyses stratifying outcomes by medication change status. Until these data are available, the observed blood pressure improvements must be interpreted as pre-post associations rather than intervention effects.
3. Participant Age Restriction and Generalizability to Older Adults: This study restricted participants to ages 60–70 years, substantially limiting generalizability to adults aged >70 years—who represent the fastest-growing and highest-risk segment of the hypertensive population. This age restriction was implemented to ensure a relatively homogeneous study population with respect to baseline digital health literacy, multimorbidity burden, functional independence, and informal caregiver support availability. However, individuals >70 years differ markedly from our sample on several dimensions that substantially influence mHealth effectiveness: First, digital health literacy declines substantially with age. Our sample (mean age 63.3 years) included early older adults with relatively preserved technology skills. By contrast, individuals >75 years have considerably lower smartphone proficiency, reduced comfort with app-based interfaces, and greater prevalence of sensory impairments (vision, hearing) that affect technology usability. Second, multimorbidity burden escalates dramatically. Our sample had 1.7 mean comorbidities; individuals >75 typically have 3–5 chronic conditions. This multimorbidity creates competing demands on health management attention, reduces medication adherence capacity, and increases risks of adverse drug–drug interactions affecting hypertension treatment. Third, functional independence is substantially reduced. Our participants (mean age 63, only 4.3% living alone) retained near-normal functional status. Individuals >75 frequently experience: (a) mild cognitive impairment (12–18% prevalence); (b) limitations in basic ADLs or instrumental ADLs (30–40%); (c) hearing or vision impairments requiring interface modifications; (d) greater dependence on family caregivers for health management and technology use. These functional limitations may necessitate substantially different implementation approaches (simplified interfaces, caregiver involvement, proactive rather than reactive outreach by family doctors). Fourth, caregiver availability and engagement patterns differ. Our cohort had high rates of family-based living (95.7%). In oldest-old populations, caregiving arrangements are often more fragile, adult children may live at distance, and caregiver burden increases substantially. These differences suggest that while the hierarchical care model’s organizational framework may have merit for oldest-old populations, the digital implementation and engagement strategies may require substantial modification. Future studies specifically targeting adults >75 years should: (a) assess baseline digital health literacy and adapt interfaces accordingly; (b) explicitly involve caregivers in implementation; (c) provide greater proactivity from family doctors rather than relying on patient-initiated engagement; (d) account for multimorbidity management complexity; and (e) measure implementation fidelity and engagement separately for this higher-risk population. Without such targeted investigation, the benefits and scalability of this model for the oldest-old population remain unknown.
4. Participant engagement and intervention fidelity were not assessed using standardized measures across all intervention components. Although platform-based blood pressure uploads were recorded, detailed information on intervention exposure, adherence to individual components, and fidelity of intervention delivery was not systematically collected. Therefore, the relationship between intervention exposure and observed changes in outcomes could not be evaluated. This limitation also prevents determination of whether differences in engagement contributed to the observed variability in outcomes.
5. HRQoL outcomes were assessed using self-reported questionnaires and may therefore have been influenced by subjective perceptions and reporting biases. In addition, the 12-month follow-up period does not allow assessment of the longer-term sustainability of the observed changes. Furthermore, specific details of blood pressure measurement procedures, including participant posture, arm positioning, and cuff size, were not prospectively recorded in the study database and could not be reliably reconstructed retrospectively. Although endpoint blood pressure measurements were obtained using a calibrated electronic sphygmomanometer by trained research personnel, these unrecorded procedural details may have introduced some measurement variability.
6. Although the retention rate was high at 93.3%, attrition bias cannot be excluded. Loss to follow-up may have been associated with health status, comorbidity, or other prognostic characteristics, and a formal comparison of baseline characteristics between participants who completed follow-up and those who discontinued was not performed in the present analysis. Therefore, the direction and magnitude of any potential attrition-related bias remain uncertain.

## 5 Conclusion

In conclusion, this prospective single-arm pre-post study suggests that a mHealth-enabled hierarchical care model integrated within regional medical consortia is feasible to implement over a 12-month period among adults aged 60–70 years with hypertension. The recruitment of 225 participants, successful completion of the 12-month follow-up by 210 participants, corresponding to a 93.3% retention rate, sustained platform engagement, and delivery of the intervention throughout the follow-up period provide preliminary support for implementation feasibility. Improvements were observed in blood pressure indicators and HRQoL, including the anxiety/depression dimension of the EQ-5D-5L. However, these changes cannot be attributed specifically to the intervention because the study did not include a concurrent control group and did not systematically capture longitudinal medication changes or detailed intervention fidelity. Controlled studies with longer follow-up and more comprehensive assessment of medication use, participant engagement, intervention fidelity, attrition, sustainability, and cost-effectiveness are warranted to determine the effectiveness and scalability of this model.

## Data Availability

The original contributions presented in the study are included in the article/supplementary material, further inquiries can be directed to the corresponding author/s.
